# Identification of genetic risk loci and prediction for β‐amyloid deposition in the East Asian population

**DOI:** 10.1002/alz.091399

**Published:** 2025-01-03

**Authors:** Jun Pyo Kim, Sang‐Hyuk Jung, Muhammad Ali, Carlos Cruchaga, Beomjin Jang, Towfique Raj, Hong‐Hee Won, Sang Won Seo

**Affiliations:** ^1^ Samsung Medical Center, Sungkyunkwan University School of Medicine, Seoul Korea, Republic of (South); ^2^ Samsung Advanced Institute for Health Sciences and Technology (SAIHST), Sungkyunkwan University, Samsung Medical Center, Seoul Korea, Republic of (South); ^3^ Perelman School of Medicine, University of Pennsylvania, Philadelphia, PA USA; ^4^ Department of Psychiatry, Washington University, St. Louis, MO USA; ^5^ Washington University in St. Louis, School of Medicine, St. Louis, MO USA; ^6^ Icahn School of Medicine at Mount Sinai, New York, NY USA; ^7^ Samsung Medical Center, Seoul Korea, Republic of (South)

## Abstract

**Background:**

Previous Alzheimer’s disease GWAS studies were mostly based on the European population, and the β‐amyloid (Aβ) status was not considered. We performed a meta‐GWAS using East Asian and European genomics data and performed prediction of Aβ status using the identified variant. We utilized single‐cell transcriptome data to identify the differentially expressed gene that is affected by the variant.

**Method:**

In our discovery dataset**,** genotype data of 3,387 participants from Korea‐Registries to Overcome and Accelerate Dementia research cohort, who underwent amyloid PET were included in our analysis. Additionally, we utilized summary statistics from a recent multi‐ethnic amyloid PET GWAS using genome data from 13 cohorts. We performed a meta‐analysis to identify risk loci for cerebral Aβ deposition. We used 606 independent Korean participants for replication and prediction of Aβ positivity. Finally, post GWAS analysis was performed using Columbia ROSMAP(N = 402) and Samsung Hospital Brain Bank (N = 9) single‐cell transcriptome data.

**Result:**

In our meta‐GWAS analysis, we identified 16 SNPs showing genome‐wide significant associations near *SORL1*. The SNP rs76490923 (MAF in East Asians = 0.21) showed the most significant association (p = 3.12⨉10^‐11^). This SNP was associated with Aβ positivity in the replication samples (OR(95% CI) = 0.71(0.52‐0.96), p = 0.025). When we stratified the replication dataset by APOE e4 carrier status and SNP rs76490923, the risk of Aβ positivity was significantly impacted in e4 carriers. (OR = 2.30 in rs76490923 homozygotes, OR = 8.41 in rs76490923 non‐carriers). The single‐cell eQTL results from Columbia ROSMAP database showed that rs76490923 was associated with the expression level of *SORL1* in microglia (p = 3.86⨉10^‐6^). Furthermore, in SMC Brain Bank autopsy cases, SORL1 was differentially expressed in microglia (logFC = ‐1.335, FDR = 3.81⨉10^‐83^) between Aβ positive and negative individuals.

**Conclusion:**

While variants related to *SORL1* have not been identified in a recent multi‐ethnic amyloid PET GWAS, our analysis including greater proportion of East Asian population identified significant SNPs near *SORL1*. This association might have been undetected in previous studies due to Europeans' low minor allele frequency (0.023). Our finding supports the importance ethnic diversity, which has been increasingly recognized recently in AD genomics studies.

